# Sonographic-Assisted Catheter-Positioning in Intracerebral Hemorrhage

**DOI:** 10.3389/fneur.2018.00651

**Published:** 2018-08-07

**Authors:** Wolf-Dirk Niesen, Matthias Reinhard, Mortimer Gierthmuehlen, Hannah Fuhrer

**Affiliations:** ^1^Department of Neurology, Medical Center-University of Freiburg, Freiburg, Germany; ^2^Department of Neurology, Medical Center Esslingen, Teaching Hospital of the University of Tuebingen, Esslingen, Germany; ^3^Department of Neurosurgery, Medical Center-University of Freiburg, Freiburg, Germany

**Keywords:** stroke, intracerebral hemorrhage, sonography, transcranial ultrasound, catheter insertion

## Abstract

**Introduction:** Intracerebral structures and pathologies such as intracerebral hemorrhages (ICH) can be displayed sufficiently by transcranial sonography (TCS). In some patients with ICH clot evacuation via surgery or catheter drainage to reduce secondary parenchymal injuries may be necessary. We hypothesized that bedside-placement of drainage-catheters, which is a minimal invasive evacuation-technique complicated by a higher rate of catheter misplacement can be optimized via TCS.

**Methods:** Eleven consecutive ICH-patients diagnosed via computertomography (CT) were included in this prospective observational pilot study. All patients were examined via TCS, firstly in order to illustrate the hematoma, secondly to optimize catheter placement. Catheter placement was primarily validated via CT.

**Results:** The TCS-depiction of ICH-extension was optimal in 10 patients; one patient showed a partially insufficient transtemporal bone window. Catheter positioning could be traced and adapted correctly via TCS-examination in all patients. Follow-up CT-scans confirmed TCS-description of catheter-positioning in all patients without any complications. Reduction of symptoms and ICH-volumes confirmed effectiveness of treatment.

**Conclusions:** The illustration of ICH and the drainage-placement is possible via TCS in a cost- and time-efficient way.

## Introduction

Intracranial hematomas occur in 10–15% of all stroke patients (1). In cases of space-occupying hematoma or intraventricular hemorrhage surgical removal or catheter drainage may be crucial in order to reduce secondary injuries due to mass effects, hydrocephalus, excitotoxic stress, and perifocal edema (1–4).

In space-occupying ICH, minimally-invasive techniques, such as stereotactic or neuronavigation-assisted hematoma evacuation via catheters, have been developed as an alternative treatment to open hematoma evacuation and decompressive hemicraniectomy (2). However, these procedures are time-consuming and are performed in an operating room. Therefore, bedside techniques on the intensive care unit can be used in non-agitated or sedated patients reducing side effects of transportation, general anesthesia, and being time-efficient(2–4). The puncture site is selected based on computertomographic data with three-dimensional planning but to date these techniques offer no concurrent visual control of catheter placement and thus are dependent on the neurosurgeons' expertise (5, 6).

Cerebral structures and pathologies such as ICH can be depicted via transcranial gray-scale sonography (TCS) with a high sensitivity and specificity comparable to those of CT-scans (7, 8). We hypothesized that TCS may illustrate the ICH correctly and facilitate optimal catheter positioning by depicting the metal stylet of the drainage catheter.

## Materials and methods

### Subjects

Eleven consecutive ICH-patients who were admitted to the Neurological Intensive Care Unit (NICU) of University Medical Center in Freiburg and in whom urgent evacuation of ICH was planned were included into this prospective observational pilot study. ICH was diagnosed via CT-scans on admission by a neuroradiologist. This study was carried out in accordance with the recommendations of the local ethic committee in accordance with the Declaration of Helsinki. As an observational and pilot study only without any treatment impact no approval of an ethics committee was needed. The catheter insertions were emergency interventions with the patients being in danger for neurological deterioration; therefore, no written consent was obtained.

### Transcranial sonography

TCS was used by experienced neurologists in order to assess intracerebral structures with a GE Logique 7 expert ultrasound system (GE Healthcare, USA) with a 2.0–2.5 MHz-transducer using the transtemporal approach in a meato-orbital plan. Visualization of hyperechogenic blood was performed progressing from the contralateral side of the lesion to a scanning-depth of 16 cm and tilting the probe to the maximum extent of the hematoma. Technical details of TCS has been outlined previously (7). A second TCS-examination was performed during catheter-insertion in order to illustrate catheter-placement by depicting the trajectory of the metal stylet of the inserted catheter from the skull opposite to the probe into the hematoma. TCS and CT data was compared descriptively regarding localization of ICH and catheter. ICH-volumes were measured before and after drainage on the CT-scans.

### Catheter placement

Catheter insertion was planned and performed by a neurosurgeon in a bedside-technique on the NICU. Patients were positioned according to puncture site and burr-hole trepanation coordinates. Target trajectory was then transferred from 3D-reconstructed CT-data to the patients' heads. Under sterile conditions a 3.5 mm burr-hole trepanation was performed using a motorized hand drill (Acculan, Aesculap, Tuttlingen, Germany) and a scaled external ventricular catheter (Spiegelberg, Hamburg, Germany) was inserted at calculated angle and depth into the center of the hematoma. After initial hematoma aspiration, the inserted catheter was attached to the skin, a sterile drainage system was connected to it and a follow up-CT was done to verify the catheter position [for a more detailed procedure description see (5, 6)]. Correct catheter position was defined as a position of the catheter tip and sideholes within the boundaries of the hematoma with a distance of at least 1 cm to the hematoma border. In case of ventricular drain correct placement was defined to international standards with localization of catheter tip with side holes within the frontal horn of the side ventricles.

### Statistics

Clinical, TCS- and CT-data was compared descriptively and via Fisher's exact test regarding localization of ICH and catheter position. ICH-volumes before and after treatment were compared with *T*-test.

## Results

Four of the 11 patients included in this study were women. Patients were 69.5 ± 9.5 years old, initial median National Institute of Stroke Scale (NIHSS) was 12 ± 7.8 points and nine patients showed clinical symptoms of ICH-mass effects with neurological decline. Three patients died during hospital stay, the surviving patients' median NIHSS at discharge was 7±3.3 points. Patients' characteristics are depicted in Table 1. Three patients received external ventricular drains; in the other patients, the drains were placed directly into the ICH-centers. Volumetric measures showed ICH-volumes with 51.6 ± 25.1 ml before catheter insertion and 22.2 ± 18.6 ml after drainage. ICH-volume was significantly reduced after treatment (*p* = 0.001).

**Table 1:** Patients' characteristics.

**Patient No**.	**ICH location**	**ICH etiology**	**NIHSS on admission**	**NIHSS at discharge**	**Outcome at discharge**
1	Right, deep parenchymal, and intraventricular	Traumatic	9	6	Transfer to rehabilitation center
2	Right, lobar (temporal), and intraventricular	Oral anticoagulation	12	10	Transfer to rehabilitation center
3	Right, deep parenchymal, and intraventricular	Hypertensive	2	2	Transfer to rehabilitation center
4	Bilateral SAH, right lobar (central)	Unknown	5	–	Death during hospital stay
5	Left, lobar (temporal), intraventricular, and SAH	Hypertensive	24	-	Death during hospital stay
6	Right, parenchymal (frontal)	Hemorrhagia of infarction	8	3	Transfer to rehabilitation center
7	Right, lobar (temporal, occipital)	Oral anticoagulation	30	–	Death during hospital stay
8	Right, deep parenchymal, intraventricular, SAH	Unknown	15	9	Transfer to rehabilitation center
9	Left, deep parenchymal, intraventricular	Hypertensive, oral anticoagulation	7	8	Transfer to rehabilitation center
10	Left, lobar (parietal), and intraventricular	Hypertensive	27	18	Transfer to rehabilitation center
11	Left, lobar (temporal)	Unknown	2	2	Transfer to rehabilitation center

*ICH, intracerebral hemorrhage; SAH, subarachnoid hemorrhage; NIHSS, National Institute of Health Stroke Scale*.

**Figure 1:**
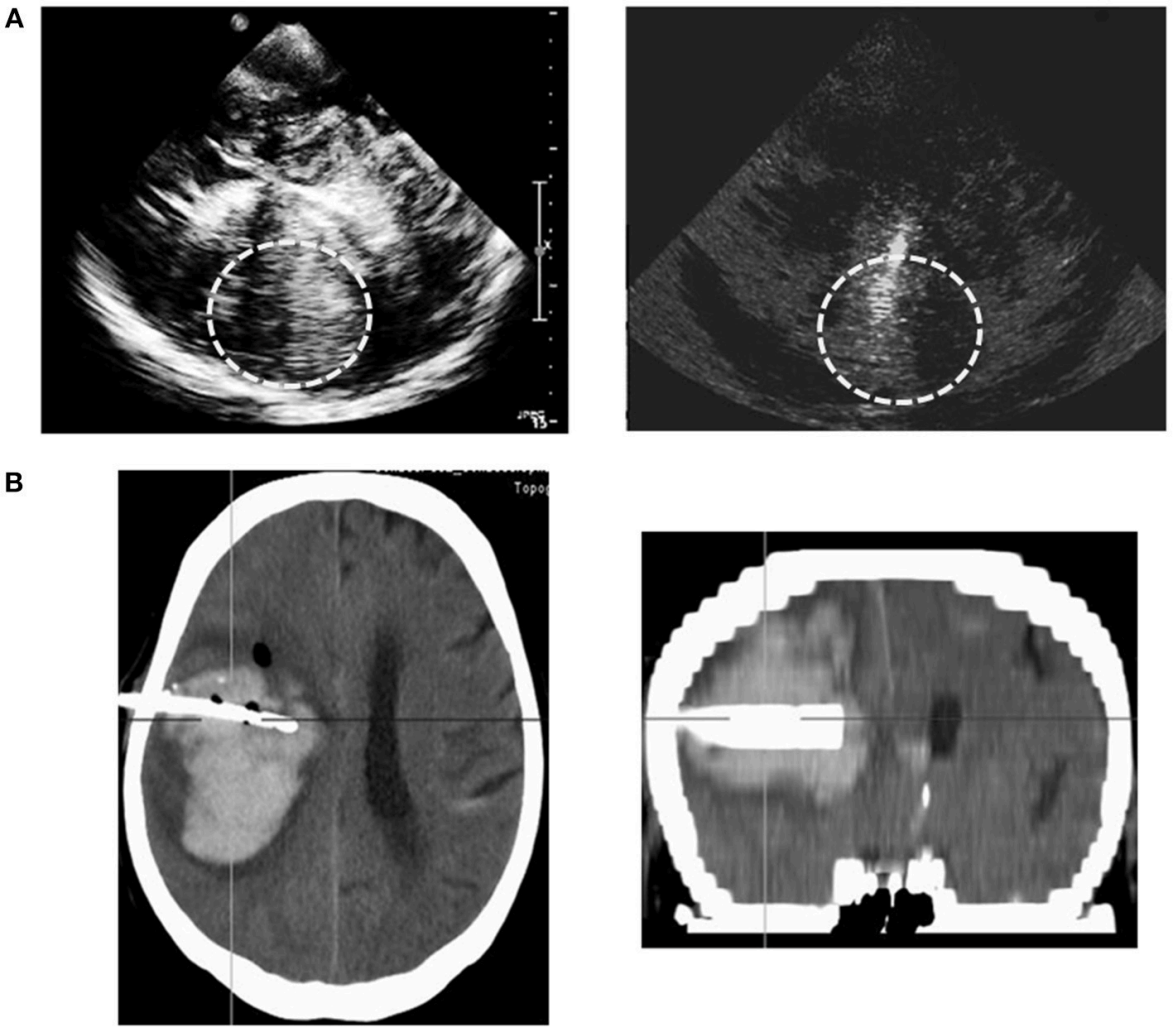
Example of sonographic illustration of hematoma and catheter positioning compared to the CT-scan. Sonographic depiction of the hematoma and inserted catheter trajectory of the metal catheter stylet (gray-scale imaging). Display of the hematoma and catheter on the CT-scan (coronal and transversal plane); planned trajectories for catheter insertion.

One patient showed an insufficient transtemporal bone window leading to a restricted ICH-illustration via TCS, while the TCS-depiction of ICH-extension was optimal in all other patients. Catheter positioning could be traced and adapted correctly via TCS-examination in all patients. An illustrative example is shown in Figure 1. Follow-up CT-scans confirmed correct catheter-positioning in 10 of 11 patients without any complications. Catheter position was detected as suboptimal with correct location in the ICH but <1 cm to the boundaries on TCS as well as on CT in one patient. The catheter was left unchanged after verifying sufficient drainage. In 10 of 11 patients TCS-description of catheter-position corresponded very well with CT-illustration; there was a slight difference of description in one patient. These descriptive results were reflected by a trend in the Fisher's exact test, χ^2^(1) = 11.00, *p* = 0.91.

## Discussion

The results of this prospective pilot study show that an optimal catheter placement with visual adjustment due to TCS in space-occupying ICH is possible. So far, all kinds of minimally-invasive techniques, stereotactic, and neuronavigation-assisted operations offer no options for visual adjustment during the procedure. Visual validation, as shown in our data, is not only possible to depict intracerebral structures (7, 8) but also the route and positioning of foreign bodies, such as catheters.

Little data is available only regarding correct catheter drainage using the bedside-technique described (5). Bedside-placement of intraventricular drainages is optimal in 81–93% (6). In our patients, all catheters were placed correctly within the hematoma or the ventricular blood clot with only one suboptimal placement in no need for catheter adjustment. Therefore, no second operation or CT-scan was needed in order to replace the catheter. Bleeding complications of bedside-procedures range from 5 to 22% (5, 6). In our cohort, no complications occurred. Clinical and CT-data confirm the effectiveness of treatment with a reduction of NIHSS-scores and ICH-volumes.

It is often pointed out that TCS is limited by insufficient transtemporal bone window (in 16–36% of the European population) (9). This was the case in one of our patients; however, sonographic-assisted catheter positioning was still possible in this patient. In general, small hemorrhages in the frontal or parasagittal region cannot be adequately assessed via TCS as the spatial resolution in these areas is limited (10). Correlation of TCS- and CT-data is high (7, 8); we believe the Fisher' exact test showing a trend only stems from the low patient number examined in this pilot study. Therefore, the results of the present study should be regarded as preliminary and further randomized controlled studies should confirm our results.

We conclude that the illustration of ICH and the drainage-placement is possible via TCS in a cost- and time-efficient way. This technique is limited in rare cases of insufficient temporal bone window and restricted spatial resolution in frontal or parasagittal areas.

## Author contributions

W-DN and MR conceptualized the study design. W-DN, MR, and MG acquired clinical data. HF analyzed the study data and drew the first draft of the manuscript. All authors reviewed and accepted the manuscript.

### Conflict of interest statement

W-DN and HF have received travel expense funding of Fresenius and AbbVie, respectively. MR has served on an advisory board for Daiichi Sankyo Inc. and has received speaker honoraria from Bayer Healthcare and Boehringer Ingelheim. MG is an advisor of Neuroloop GmbH.
